# Healthy Baby Delivery After 100 Days of Somatic Support in a Brain-Dead Pregnant Woman

**DOI:** 10.1155/crog/2746188

**Published:** 2025-05-21

**Authors:** Luísa Cerqueira, José Manuel Pereira, Marina Moucho

**Affiliations:** ^1^Obstetrics Department, Centro Hospitalar Universitário de São João, Porto, Portugal; ^2^Intensive Care Medicine Department, Centro Hospitalar Universitário de São João, Porto, Portugal

## Abstract

After diagnosing brain death, with few exceptions, it is unethical to maintain organ support. This decision may be considered in case of pregnancy to guarantee foetal viability and improve the perinatal outcome. We describe a case of prolonged organ support after brain death in a pregnant woman, following cardiac arrest secondary to an acute asthma attack. A 26-year-old primigravida, 18 weeks pregnant, with a history of stable asthma, was admitted to an ICU after cardiac arrest secondary to an acute asthma attack. It was possible to maintain organ support for 100 days with the collaboration of a multidisciplinary medical team. A healthy child was born, and favourable outcomes were observed at the 3-year follow-up. Brain death during pregnancy is a rare event and is a unique ethical, financial, and technical challenge. No guidelines are available for the management of these patients. This case demonstrates that it is possible to prolong organ support in this situation and successfully deliver a healthy child.

## 1. Introduction

Prolonging somatic support after brain death (BD) is considered unethical, unless to allow organ donation or during pregnancy, in order to improve perinatal outcomes [1].

Brain death during pregnancy is a rare event with less than 40 cases of somatic support prolongation described in the literature, with 28 neonates being born alive [1, 2]. When facing such situations, a dilemma is met: Should the delivery be immediate and the woman's somatic support be discontinued, or should an attempt to prolong pregnancy in order to achieve foetal maturity be made? [3].

We report a case of prolongation of somatic support after BD in an 18-week pregnant woman and the successful delivery of a healthy child after 100 days of maternal somatic support.

## 2. Case Report

A 26-year-old primigravida, 18 weeks pregnant, with a history of stable asthma treated with inhaled bronchodilators and an uncomplicated course of pregnancy, presented with progressive dyspnoea, without cough, sputum, or fever, and collapsed at home. Basic life support was started immediately by a family member, and 20 min later, she was found by first responders in cardiac arrest with no shockable rhythm. After 15 min, the medical team confirmed cardiac arrest with pulseless electrical activity, which returned to spontaneous circulation after two cycles of advanced life support. She was intubated at the scene and then admitted to an intensive care unit (ICU) for postresuscitation care with a presumptive diagnosis of cardiac arrest secondary to an acute asthma attack.

**Figure 1:**
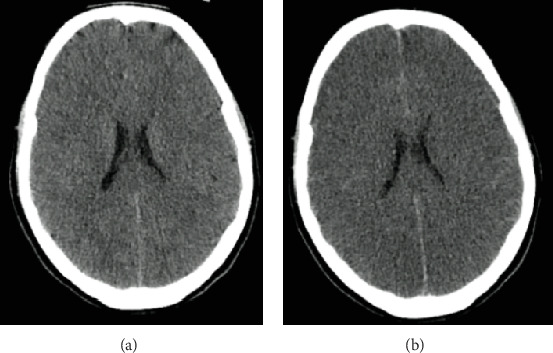
CT head scan at admission to the hospital (a) and at 48 h (b). On the right, the image shows diffuse hypodensity of cerebral hemispheres suggestive of ischemia as well as sulci loss and ventricular system reduction indicative of diffuse cerebral oedema.

On Day 3, there were no signs of neurological recovery despite minimal sedation. The CT head scan showed diffuse hypodensity of cerebral and cerebellar hemispheres and brainstem suggestive of ischemia as well as diffuse cerebral oedema (Figure 1). Sedation was held on Day 6, and the patient was then submitted to BD testing. BD was confirmed according to the hospital protocol after 7 days in the ICU.

Taking into account the family's wishes, a statement was requested to the ethics committee to proceed with somatic support in order to prolong pregnancy. After its approval, a multidisciplinary medical team decided to maintain somatic support of the brain-dead woman, the key aim being the delivery of a healthy child at 32 weeks of gestational age. A weekly meeting including the ICU, obstetrics, and neonatal departments, a psychologist, and an ethics committee took place in order to inform and involve the family members.

Standard monitoring of vital signs and daily laboratory testing was performed during the ICU stay. During the course of hospitalization, a lung-protective mechanical ventilation strategy was favoured with FiO_2_ requirements between 35% and 50% and PaO_2_/FiO_2_ ranging from 250 to 275. Low-dosage vasopressors (noradrenaline between 0.03 and 0.15 *μ*g/kg/min) were needed to maintain the mean arterial pressure> 75 mmHg. No further invasive organ support was needed during the ICU stay. Enteral nutrition was used to provide the total daily calorie requirement.

Throughout the ICU stay, several complications occurred, the most relevant being endocrinological dysfunctions, secondary to hypoxic brain injury, and respiratory and urinary infections (Table 1). Panhypopituitarism was documented, requiring levothyroxine, desmopressin, and fludrocortisone supplementation.

Gynaecology and obstetrics consultants managed the course of pregnancy. Until foetal viability was reached, routine exams were performed, including laboratory testing and the midtrimester foetal ultrasound scan which found no major malformations. A course of dexamethasone for foetal lung maturity was administered at 24 weeks of pregnancy. After this gestational age, the pregnant woman was transferred to a different hospital to ensure that the ICU, obstetrics, and neonatal care were in the same building.

From this point onwards, foetal heart rate was assessed with ultrasound on a daily basis, and after 28 weeks, daily cardiotocography was used. Obstetric ultrasound was performed every 2 weeks to assess foetal growth and biophysical profile. At 30 weeks, a foetal growth restriction was diagnosed, with an estimated foetal weight of 1295 g (7th centile) and a middle cerebral artery pulsatility index and a cerebroplacental ratio below the 5th centile. A brain foetal magnetic resonance imaging was performed at 31 weeks, and no apparent central nervous system anomalies were detected. A rescue course of dexamethasone was administered at this point, 1 week before the scheduled pregnancy termination (32 weeks).

At 31 weeks and 6 days, 100 days after admission, the pregnant woman developed sudden hypoxia caused by left lung atelectasis that did not resolve even after bronchoscopy. Considering the maternal worrying condition and the cardiotocography trace showing reduced variability, the decision to deliver the child by urgent caesarean section was made, and the organ-transplant protocol was initiated.

A male infant was delivered weighing 1700 g and with an Apgar score of 2, 4, and 8 at 1, 5, and 10 min, respectively. The child spent 40 days in the ICU developing typical complications of prematurity, including mild respiratory distress syndrome and retinopathy of prematurity stage 1. He was ventilated for 14 h, and then, noninvasive ventilation was needed for 14 days. One dose of surfactant was used to treat the respiratory distress syndrome. At 3 years of follow-up, the child's development was normal, and no apparent sequelae were identified.

## 3. Discussion

Specific legal and ethical issues must be taken into account when deciding whether or not to continue somatic support of a pregnant woman after the diagnosis of BD. In Portugal, since there is no legislation concerning this subject, the decision should, after consideration of a living will and/or the family's wishes, be approved by an ethics committee. Although not yet published by the time the reported case occurred, in retrospect, the International Federation of Gynaecology and Obstetrics guidelines for the management of the social and ethical challenges in BD during pregnancy were fulfilled [4]. In this particular case, the low gestational age was imperative to consider, since it demanded a longer organ support duration, imposing a greater financial and emotional investment, with no guarantee that a healthy infant would be born.

Dodaro et al. reported that the main causes of BD in pregnant women were intracranial haemorrhage, subarachnoid haemorrhage, or hematoma (68%), trauma (12%), suicide attempt (9%), and cerebral tumour or mass (6%) [1]. Another vital consideration, in this case, was regarding the cause of BD, which was an acute asthma attack that was severe enough to cause cardiac arrest and hypoxic brain injury to the mother and increase the uncertainty about the possible impact of this aggression on the foetus.  Table 2 reviews the available cases in literature of brain-dead pregnant women after cardiac arrest.

An effort was made to maintain the pregnancy until viability was reached. But since neonatal mortality is substantial even at 24 weeks of gestational age (32.1%), it was established that 32 weeks would be the gestational age goal considering that by then, the neonatal mortality is much lower: 1.2% [8]. Another consideration was the risk of neurodevelopmental disability, which is 23.9% in extremely preterm infants compared with 4.7% in moderately preterm infants [9, 10].

We managed to maintain the mother on somatic support for 100 days, superior to the mean time reported by Dodaro et al. of 48.9 ± 38.8 days [1]. Reinhold et al. reported the most extended critical care treatment in this scenario with a total duration of 151 days [11]. To our knowledge, there are only other four cases described in the literature in which the somatic support surpassed 100 days [1, 11]. See Table 3 for a summary of cases of brain-dead pregnant women with more than 100 days of somatic support.

Considering the rarity of the situation, there are no available guidelines for the management of pregnant brain-dead patients. Our management was mainly based on our experience with other brain-dead patients. According to the guidelines on the management of potential organ donors in the ICU of the Society of Critical Care Medicine (2015), cardiovascular instability requiring hemodynamic monitoring and support and endocrine dysfunction requiring hormonal replacement therapy (vasopressin or analogues, corticosteroids, thyroid hormones, and insulin) are two fundamental aspects of managing brain-dead patients [15]. Other important interventions include protective mechanical ventilation, nutritional and electrolyte support, temperature regulation due to the risk of hypothermia, and prevention and treatment of infectious complications [16].

In pregnant brain-dead patients, complications have been described and are similar to those of other brain-dead patients. Long-term management of pregnant brain-dead patients may be associated with infections (69%), circulatory instability (63%), diabetes insipida (56%), thermal variability (41%), and panhypopituitarism (34%) [1]. The overall management of these patients should be in line with recommendations for other brain-dead patients [2]. However, a few exceptions should be taken into consideration. First, a physiological hypocarbia should be maintained during mechanical ventilation to facilitate the elimination of foetal carbon dioxide. Maternal carbon dioxide tensions should be maintained between 28 and 31 mmHg. Second, an appropriate nutritional intake should be calculated based on maternal serum alimentary values, the weight of the mother, and foetal growth. Notably, a pregnant brain-dead woman expends 75% of a healthy pregnant woman's basal energy expenditure [17]. Third, pregnant brain-dead patients usually require a longer period of somatic support, which may increase the risk of adverse events related to invasive devices, such as physical injuries or nosocomial infections [18]. Finally, these patients require regular foetal monitoring to guide obstetrical care and to define the optimal delivery strategy, which should be provided by experienced obstetrics clinicians and nurses. The most common indications for delivery are frequently associated with maternal complications resulting in cardiocirculatory instability (38%) and/or nonreassuring foetal testing (35%) [1].

## 4. Conclusions

Brain death during pregnancy is a rare event and is accompanied by a unique ethical, financial, and technical challenge. Although maternal BD is always a high-risk scenario for foetal development, in this particular case, we have to consider the long support duration, superior to the mean time reported in the literature, and the cause of the BD of the pregnant woman, which was an acute asthma attack that was severe enough to cause cardiac arrest and hypoxic brain injury in the mother and increase the uncertainty about the possible impact of this aggression on the normal development of the foetus.

## Figures and Tables

**Table 1:** Infectious complications during ICU stay.

**GA | day**	**Infection**	**Agent**	**Treatment**
18 weeks + 5 days | D5	Tracheobronchitis	Unknown	Amoxicillin & clavulanic acid (10 days)
20 weeks + 5 days | D18	UTI	*Escherichia coli*	Already under treatment for a respiratory infection when the result was known
21 weeks + 1 day | D22	Ventilator-associated pneumonia	MSSA + *Pseudomonas aeruginosa*	Piperacillin & tazobactam plus vancomycin (3 days) followed by amoxicillin & clavulanic acid (10 days), followed by piperacillin & tazobactam (10 days)
24 weeks + 1 day | D44	Tracheobronchitis	*Pseudomonas aeruginosa*	Meropenem (7 days)
25 weeks + 2 days | D54	UTI	*Pseudomonas aeruginosa*	Meropenem (7 days)
30 weeks + 1 days | D88	UTI	*Pseudomonas aeruginosa*	Meropenem (7 days)

Abbreviations: D: day; GA: gestational age; MSSA: methicillin-sensitive *Staphylococcus aureus*; UTI: urinary tract infection.

**Table 2:** Summary of available cases in literature of brain-dead pregnant women after cardiac arrest [1, 5–7].

**Reference, year**	**Maternal age**	**Cause of BD**	**GA at BD (weeks)**	**Time from BD to delivery (days)**	**GA at birth (weeks)**	**Birthweight (g), AS at 5 min**	**Infant outcome**
Holliday and Magnuson-Woodward, 2017 [5]	36	HBI after cardiac arrest secondary to NSTEMI	19	90	31	1635 g, NA	Typical complications of prematurity; healthy at 1 month when discharged from NICU
Nishimura et al., 2016 [6]	30	HBI after cardiac arrest secondary to suicide attempt	23	3	24	690 g, NA	Serious neurologic disability
Kinoshita et al., 2014 [7]	32	HBI after cardiac arrest secondary to suicide attempt	20	78	33	2130 g, 8	Healthy after 40 days in NICU

Abbreviations: AS: Apgar score; BD: brain death; GA: gestational age; HBI: hypoxic brain injury; NA: not available; NICU: neonatal intensive care unit; NSTEMI: non-ST elevation myocardial infarction.

**Table 3:** Summary of available cases in literature of brain-dead pregnant women with more than 100 days of somatic support [1, 11–14].

**Reference, year**	**Maternal age**	**Cause of BD**	**GA at BD (weeks)**	**Time from BD to delivery (days)**	**GA at birth (weeks)**	**Birthweight (g), AS at 5 min**	**Infant outcome**
Reinhold et al., 2019 [11]	28	ICH after traffic accident	9	151	30	NA, 8	Typical complications of prematurity; healthy at 7 weeks when discharged from NICU; healthy at 12 months on follow-up
Said et al., 2013 [12]	35	ICH	16	110	32	750 g, 7	Born alive
Spike, 1999 [13]	20	ICH	16	100	31	1440 g, 8	8 weeks in NICU with no unusual complications
Bernstein et al., 1989 [14]	30	Traumatic brain injury after traffic accident	15	107	32	1555 g, 9	Healthy at 11 months

*Note:* ICH: intracranial haemorrhage.

Abbreviations: AS: Apgar score; BD: brain death; GA: gestational age; NICU: neonatal intensive care unit.

## Data Availability

The data that support the findings of this study are available on request through the authors themselves.
